# Quantitative and Qualitative Segmental Surface Growth in Infants with Unilateral Cleft Lip and Palate: A Prospective In Vivo Study

**DOI:** 10.3390/medicina61071232

**Published:** 2025-07-08

**Authors:** Sarah Bühling, Cedric Thedens, Sara Eslami, Nicolas Plein, Iulia Dahmer, Babak Sayahpour, Lukas Benedikt Seifert, Robert Sader, Stefan Kopp

**Affiliations:** 1 Department of Orthodontics, Johann-Wolfgang Goethe University, 60596 Frankfurt, Germany; thedens@med.uni-frankfurt.de (C.T.); eslamishahrbabaki@med.uni-frankfurt.de (S.E.); plein@med.uni-frankfurt.de (N.P.); kopp@med.uni-frankfurt.de (S.K.); 2 Institute of Biostatistics and Mathematical Modeling, Johann-Wolfgang Goethe University, 60629 Frankfurt, Germany; dahmer@med.uni-frankfurt.de; 3 Clinic for Oral, Maxillofacial, and Facial Surgery, University Hospital Basel, 4031 Basel, Switzerland; lukasbenedeikt.seifert@usb.ch; 4 Clinic for Maxillofacial and Plastic Surgery, Johann-Wolfgang Goethe University, 60629 Frankfurt, Germany; r.sader@em.uni-frankfurt.de

**Keywords:** cleft lip palate, growth analysis, prospective study, surface, maxilla, qualitative assessment

## Abstract

*Background and Objectives*: Patients with unilateral cleft lip and palate (UCLP) require a phase of infant orthopedic treatment prior to surgical cleft closure. Treatment planning in this phase necessitates a thorough understanding of maxillary growth dynamics in this period. The aim of the present study was to evaluate the quantitative and qualitative surface growth of maxillary segments in infants with UCLP. *Materials and Methods*: In total, 195 intraoral scans from 50 patients were obtained postnatal (T0), at monthly intervals (T1–5), and prior to surgical cleft closure at 6 months of age (T6). Surface, linear, and angle measurements of the maxillary segments were performed. *Results*: Significant increases in the total surface area and the surface areas of the small and large segments were observed at monthly intervals and over the overall duration. The large segment showed greater absolute growth (11.62 mm^2^ per month, 46.57 mm^2^ total), while the small segment had a higher percentage increase (1.49% monthly, 6.57% overall). A positive small correlation was observed between surface area growth changes in the small segment and its increase in length. *Conclusions*: Our results revealed distinct growth patterns of the large and small segments in amount and direction, underscoring the relevance of incorporating segment arch width in surface evaluations.

## 1. Introduction

Unilateral cleft lip and palate (UCLP) is among the most common congenital craniofacial anomalies, affecting one in every 800 live births globally, depending on geographic, ethnic, and genetic factors [1,2,3]. UCLP may involve the lip, alveolus, and palate to variable degrees. Beyond aesthetic concerns, UCLP is associated with significant functional challenges, including difficulties in feeding, chewing, breathing, speech development, and hearing for affected individuals and their families [4]. Early intervention and precise treatment planning are essential to address these challenges effectively, culminating in the surgical closure of the hard and soft tissue cleft that aims to restore the full functionality of the stomatognathic system, including improved feeding, speech, and overall oral health.

Beginning at birth, newborns with UCLP typically receive orthodontic treatment as part of presurgical infant orthopedics (PSIO) until surgical cleft closure. PSIO involves the use of individually designed plates, which serve dual purposes: they separate the nasal and oral cavities, facilitating drinking as well as swallowing, and by empirically blocking out in the cleft area they allow the cleft poles to approach each other, promoting the formation of harmonious dental arches before the cleft is surgically closed [5]. Due to ongoing growth, these feeding plates must be regularly adjusted or replaced to accommodate the infant’s changing maxillary anatomy. The effective application of PSIO in the early treatment of newborns with UCLP demands both specialized professional expertise and a thorough understanding of maxillary growth dynamics in this period.

Traditionally, the evaluation of morphological changes in the maxilla during treatment has relied on plaster casts [6,7,8,9,10] and photographs [11] of dental models. However, these techniques often suffer from inaccuracies, particularly in landmark positioning and the subsequent data transfer to digital platforms [12]. While traditional analyses of neonatal edentulous jaws with UCLP have focused on two-dimensional [6,8] or three-dimensional landmark registration on the mucosal surface [9,10,13], these approaches may not fully capture the complexity of craniofacial structures. To address these limitations, new measurement techniques, such as digital intraoral scanning and surface-based analyses, have been introduced as a more precise method for evaluating growth and treatment effects [14,15,16]. Existing surface analysis studies primarily rely on measurements of digitized plaster models and only depict the surface change for individual points in time several months apart [2,12,17,18]. In most studies investigating the surface measurement of the maxilla, the alveolar ridge acts as the border of the palate, thereby neglecting the vestibular tooth germ-bearing alveolar parts located vestibular to the palatal gingival grooves [19,20,21]. Previous studies have primarily concentrated on the overall surface area increase during the treatment period, with limited attention paid to the quality of growth—specifically, the directional development of jaw segments in the sagittal, transverse, or combined dimensions. However, during infancy, longitudinal studies of maxillary growth are limited and the amount and direction of maxillary growth at different stages of early development remain largely unknown. A thorough understanding of the extent and direction of early maxillary growth is crucial for effective PSIO treatment in newborns with UCLP. This knowledge can improve rehabilitation protocols and advance digital planning for PSIO.

The present study aims to assess the quantitative surface changes in UCLP jaw segments throughout the PSIO treatment period, as well as the monthly surface changes, offering valuable insights for digital forward planning in PISO. Additionally, new insights into the quality of the surface changes in jaw segments are to be gained by including segment length measurements as well as segment width measurements of the jaw segments within the scope of the investigation.

## 2. Materials and Methods

### 2.1. Study Design and Ethics

This monocentric prospective observation study aims to evaluate the quantitative and qualitative surface changes in jaw segments in patients with UCLP and was approved by the ethics committee of the medical department of J. W. Goethe University Frankfurt am Main (Nr. 2023-1250). Informed consent from the parents and legal guardians of all patients was obtained.

### 2.2. Patients

Fifty newborn babies (40 male, 10 female) with complete UCLP (33 left, 17 right) were consecutively enrolled in this prospective study between 1 May 2018 and 1 May 2024.

### 2.3. Inclusion Criteria

The study included male and female patients who presented with a complete unilateral cleft of the lip, alveolus, and palate (UCLP) and who had undergone presurgical infant orthopedic treatment (PSIO) with maxillary plates and surgical cleft closure at of J. W. Goethe University Frankfurt am Main. Only patients with clearly identifiable surfaces on the intraoral scans were included.

### 2.4. Exclusion Criteria

Patients with systemic diseases, syndromes, or other deformities were excluded from the study.

### 2.5. Sample Size Calculation

The sample size calculation was performed in collaboration with the Institute for Biostatistics and Mathematical Modeling of J. W. Goethe University Frankfurt am Main. The primary aim of the study was to investigate the changes in surface area between birth (T0) and just before surgical cleft closure at 6 months of age (T6). The planned analysis was a one-sample *t*-test. A number of 50 participants was sufficient for detecting an effect size (Cohen’s d) of 0.5 with at least 90% power when performing a one-sample *t*-test at a 5% significance level. A similar effect size was observed in the study by De Menezes et al. [12]. This corresponded to their estimates of 32.7 mm^2^ and 36.8 mm^2^ for the means of the minor segment surface at birth and just before OP, respectively, assuming a standard deviation for the change in the surface of 7.8 mm^2^.

### 2.6. Data Collection

**Figure 1:**
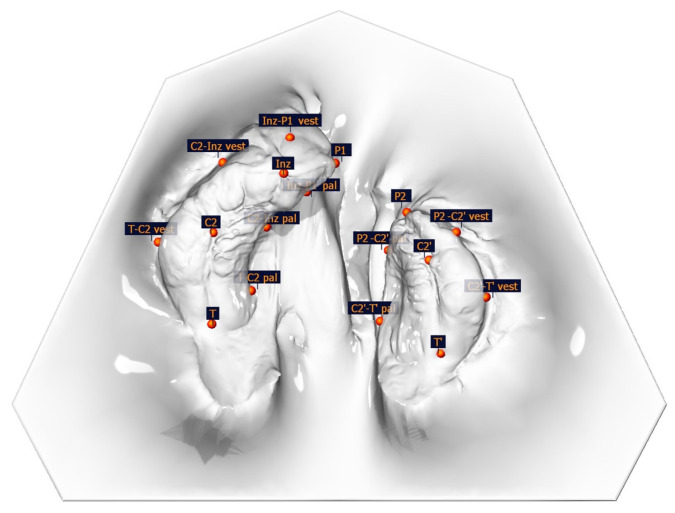
Illustration of anatomical measuring points.

**Figure 2:**
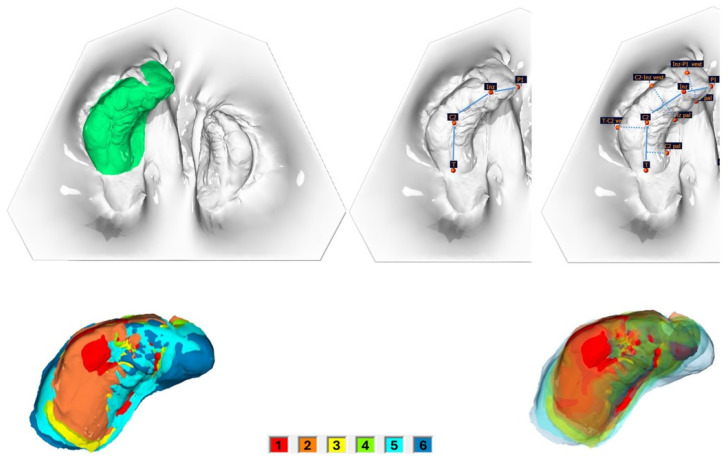
Large segment. Top left, surface measurement; top middle, segment length measurements; top right, segment width measurements. Bottom: surface enlargement of the large segment over the entire preoperative treatment period (6 IOS; 1 = after 1 month; 2 = after 2 months; 3 = after 3 months; 4 = after 4 months; 5 = after 5 months; 6 = after 6 months), without transparency (**left**) and with increasing transparency (**right**), in ascending order.

**Figure 3:**
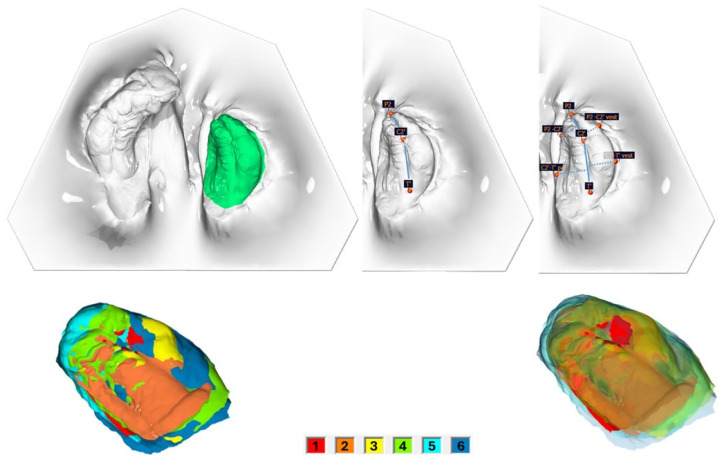
Small segment. Top left, surface measurement; top middle, segment length measurements; top right, segment width measurements. Bottom: surface enlargement of the large segment over the entire preoperative treatment period (6 IOS; 1 = after 1 month; 2 = after 2 months; 3 = after 3 months; 4 = after 4 months; 5 = after 5 months; 6 = after 6 months), without transparency (**left**) and with increasing transparency (**right**), in ascending order.

In total, 195 intraoral scans (IOS) from the babies’ upper jaw segments were obtained using a TRIOS 4 wireless intraoral scanner (3shape, Copenhagen, Denmark). Scans were taken 1–2 days after birth (T0), during the monthly therapy controls (T1 = after 1 month; T2 = after 2 months; T3 = after 3 months; T4 = after 4 months; T5 = after 5 months), and just prior to surgical cleft closure at 6 months of age (T6). Surface, linear, and angle measurements of the maxillary segments were performed on the digital dataset using OnyxCeph^3TM^ software (Version 3.2, Image Instruments, Chemnitz, Germany) by one examiner. The surface measurement boundary lines and the measurement points are shown and defined in Figure 1, Figure 2 and Figure 3 and Table 1.

### 2.7. Statistical Analysis

The statistical evaluation was carried out under the supervision of the Institute for Biostatistics and Mathematical Modeling of the Faculty of Medicine at J. W. Goethe University Frankfurt am Main. All statistical analyses and graphical representations were performed using R version 4.4.1 (R Foundation for Statistical Computing, Vienna, Austria) and RStudio (Version 202.04, Posit Software, PBC, Boca Raton, FL, USA).

Descriptive statistics, including mean value (MV), median (MD), standard deviation (SD), and interquartile range (IQR), were calculated to summarize the data. For normally distributed data, the mean and standard deviation were reported; otherwise, the median and IQR were used.

The normality of the data was checked using the Shapiro–Wilk test. Comparisons of normally distributed data were performed using the *t*-test (paired *t*-test when data were paired and the two-sample *t*-test otherwise). For non-normally distributed data, the Wilcoxon matched pairs test and Wilcoxon–Mann–Whitney U-test were used, respectively. The Pearson correlation test was performed to analyze correlations between surface and length or width measurements, and Pearson’s correlation coefficient r was computed. Pearson’s correlation coefficient r was interpreted according to Cohen [22] (r = 0.20, small; r = 0.40, moderate; and r = 0.60, large).

The significance level for all statistical tests was set at 5%.

## 3. Results

### 3.1. Quantitative Analysis

**Figure 4:**
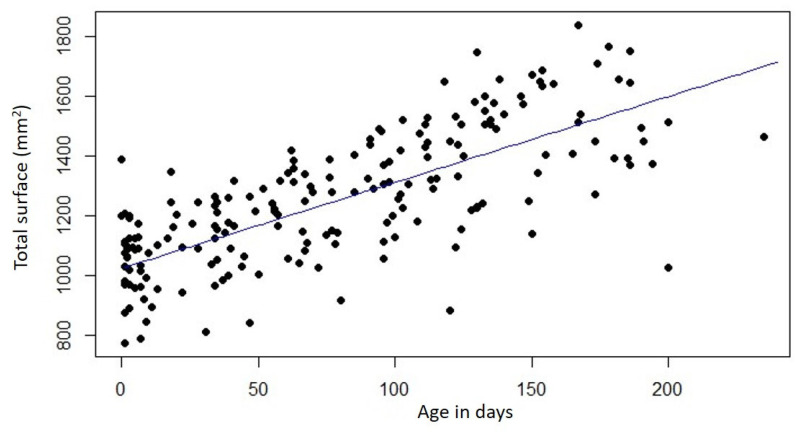
Surface measurement of total surface (small and large segment) over the PSIO treatment period.

A total of 195 surface measurements were included. The number of measurements per patient varied between 1 and 7, with a median of 4 measurements per patient. The descriptive data for the increases in the surface area of the jaw segments (total, small, large segment) are shown in Table 2. The total surface area as well as the surface areas of the small and large segments increased significantly both at monthly intervals and over the entire therapy period (*p* < 0.001) (Table 2, Figure 4). Meanwhile, the surface area of the small segment increased on average 38.69 mm^2^ per month (158.33 mm^2^ over the whole treatment period), a monthly percentage change of 9.50% (39.00% overall change). Correspondingly, the large segment’s surface area increased on average 50.32 mm^2^ (204.90 mm^2^ overall), corresponding a monthly percentage change of 8.01% (overall 32.43%). In terms of pure surface area gain in mm^2^, the large segment gained more on average per month (11.62 mm^2^) and over the entire treatment period (46.57 mm^2^) than the small segment (*p* < 0.001). However, when analyzing the percentage changes, the monthly percentage increase in the surface area of the small segment exceeded that of the large segment by 1.49% and over the entire treatment period (6.57% higher). No significant influence of cleft side (left, right) on the surface changes in the segments could be observed. Analyzing the impact of gender on segmental surface changes revealed that only the percentual monthly increase in the surface area of the large segment was significantly higher in male patients (*p* = 0.04). No significant gender differences were observed for any other measurements.

Table 3 displays the monthly changes in the total area, as well as of the small and large segments, both relative to the first measurement and in comparison to the area measurements from the preceding month. Month 7 was excluded from the analysis due to an insufficient number of cases. Except for the surface area measurement of the small segment in the 6th month, where no significant change was observed due to a very low sample size of three, all monthly surface area changes—both compared to the first month and the previous month—were statistically significant.

### 3.2. Qualitative Analysis

The quantitative analysis (Table 2 and Table 3) showed significant monthly increases in the surface areas of both jaw segments in UCLP during the preoperative treatment period. The following analysis examines the qualitative changes in the jaw segment surfaces, focusing on the transversal and sagittal measurements and their correlations.

#### 3.2.1. Transversal Changes Between Segments

To qualitatively assess the quantitative increase in surface area, we analyzed the changes in transverse parameters of both jaw segments to each other, specifically the cleft width (Table 4) and the anterior and posterior arch widths (Table 5).

The cleft width was significantly reduced during the PSIO period (*p* < 0.001). Cleft side and patient gender had no significant effect on cleft width reduction. The use of active plates resulted in a significantly greater reduction in cleft width than the use of passive plates (*p* = 0.04). Regarding the anterior and posterior alveolar arch width, no significant change could be determined during the PSIO period. Cleft side, gender, and plate type had no significant influence on transversal width change.

#### 3.2.2. Segmental Length and Width of the Segments

The segmental alveolar ridge lengths of the large segment (mesial, medial, and distal) and the small segment (mesial and distal) increased significantly over the treatment period (*p* < 0.001) (Table 6, Figure 2 and Figure 3). Cleft side and patient gender had no significant influence. In the patient group treated with active plates, the total alveolar ridge length of the small segment increased significantly more than in the passive plate group (AP 1.24 mm, PP 0.70 mm, *p* = 0.006).

The segmental alveolar ridge width of the large segment (mesial, medial, and distal) and the small segment (mesial and distal) increased significantly over the treatment period (*p* < 0.001) (Table 6, Figure 2 and Figure 3). Cleft side, patient gender, and plate type had no significant influence.

#### 3.2.3. Correlation Between Surface and Segmental Length and Width

Correlations between the increase in surface area during the PSIO treatment period and the growth in segment length and width were analyzed (Table 7 and Table 8, Figure 2 and Figure 3).

**Figure 5:**
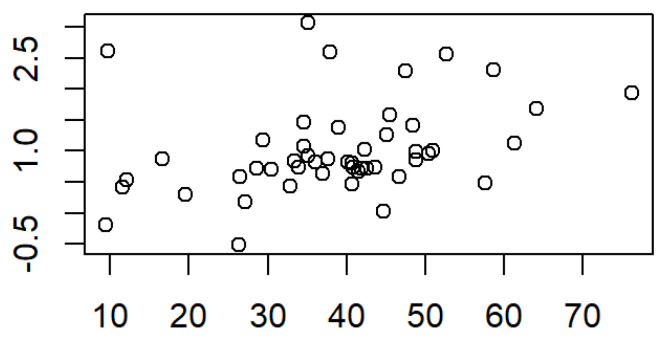
Correlation between the small segment surface and its length; r = 0.32, *p* = 0.02.

A positive significant correlation (r = 0.32, *p* = 0.02) was found between the monthly increase in surface area of the small segment (mm^2^ and percentage) and the increase in length of the small segment (Figure 5).

According to Cohen, the correlation was small. In left-sided UCLP, a weak positive correlation (r =0.004, *p* = 0.98) was observed between the increase in surface area and the increase in length of the small segment, though this correlation was not significant. By contrast, right-sided UCLP showed a large significantly positive correlation between these two variables (r = 0.65, *p* = 0.004). No gender-specific influence on the correlation could be determined. Regarding the correlation between the small segment monthly surface change and its width changes, no significant correlation could be found. Only in patients with left-sided UCLP could a positive correlation between surface and width growth be proven (r = 0.39, *p* = 0.02, small correlation).

**Figure 6:**
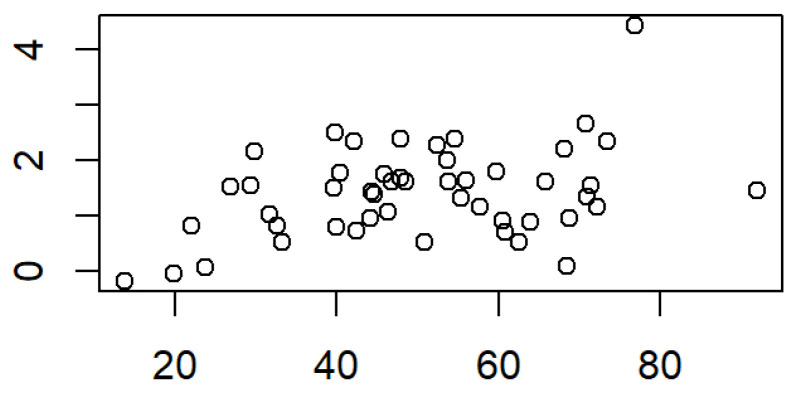
Correlation between the large segment surface and its length; r = 0.36, *p* = 0.009.

For the large segment, a positive small correlation (r = 0.36, *p* = 0.009) was found between the percentual monthly surface change and the length change (Figure 6). For the monthly overall surface change, no significant correlation with cleft side or patient gender could be detected. By contrast, there was a small significantly positive correlation (r = 0.36, *p* = 0.009; r = 0.42, *p* = 0.002) between the surface growth of the large segment and its width change, regarding both monthly overall and percentual changes (Figure 6). Moreover, a large significantly positive correlation could be proven in patients with right-sided UCLP (r = 0.61, *p* = 0.008) and male gender (r = 0.44, *p* = 0.004).

### 3.3. Angle Measurements

The angle measurements are presented in Table 9. The angle MIA describes the midpoint deviation of Inzisale. A significant reduction was observed in both the total monthly values and the percentile values (*p* < 0.001). The smaller the angle MIA, the less the incisal point deviates from the constructed point M, and the less Inzisale deviates from the center. The angle C2-T-T′ decreased significantly monthly (*p* = 0.03) and in percentage (*p* = 0.04) within the PSIO period. The angle C2-Inz-P1 decreased significantly monthly (*p* = 0.003) and in percentage (*p* = 0.004). Regarding the small segment, the angle P2-C2′-T′ decreased more significantly in patients treated with active plates than in patients treated with passive plates (*p* = 0.01). There were no significant differences in cleft side, patient gender, or plate type for all other angle changes.

## 4. Discussion

Our results showed that the total surface area as well as the surface areas of the small and large segments of patients with UCLP increased significantly over the PSIO therapy period (*p* < 0.001). While the large segment exhibited greater absolute surface area growth, averaging 11.62 mm^2^ per month and 46.57 mm^2^ over the entire treatment period (*p* < 0.001), the small segment showed a higher percentage increase, with a monthly growth of 1.49% and an overall treatment period increase of 6.57%. Gender-specific differences in the large segment’s percentage growth were deemed clinically irrelevant due to the predominance of male cases in our study sample.

This is the first study to provide a detailed month-by-month analysis of total surface area changes, as well as those of the small and large segments, both in comparison to the initial measurements and to the prior month’s data. These findings establish a valuable basis for forward planning in feeding plate production, assisting specialized centers treating patients with UCLP.

In analyzing qualitative changes, cleft width significantly decreased during the PSIO period (*p* < 0.001), with active plates achieving a greater reduction than passive plates (*p* = 0.04). No significant changes were observed in the anterior or posterior alveolar arch widths, nor did cleft side, gender, or plate type significantly influence transverse width changes. These findings suggest that jaw segments likely grew in length or width or shifted toward the cleft area as their surface area increases during PSIO therapy, while the anterior and posterior alveolar arch widths remained largely unchanged.

Alveolar ridge length and width for both the large and small segments increased significantly across all measured parts (mesial, medial, and distal) over the treatment period (*p* < 0.001). Patients treated with active plates experienced a greater total alveolar ridge length increase in the small segment compared to those treated with passive plates (AP: 1.24 mm, PP: 0.70 mm, *p* = 0.006).

Correlation analyses revealed distinct patterns. A small positive correlation was observed between the monthly and overall surface area increase and length increase of the small segment (r = 0.32, *p* = 0.02), particularly in right-sided UCLP cases (r = 0.65, *p* = 0.004). No significant correlation was found between surface area and width increases for the small segment, except in left-sided UCLP cases, where a small positive correlation was identified (r = 0.39, *p* = 0.02). For the large segment, a small positive correlation was noted between percentage surface changes and length changes (r = 0.32, *p* = 0.02). By contrast, a small positive correlation between surface and width growth was observed regarding both monthly overall and percentual changes (r = 0.36, *p* = 0.009; r = 0.42, *p* = 0.002), with stronger correlations in right-sided UCLP cases (r = 0.61, *p* = 0.008) and male patients (r = 0.44, *p* = 0.004).

Rotation analyses revealed a significant internal rotation of the large segment toward the center, evidenced by significant reductions in the MIA, C2-T-T′, and C2-Inz-P1 angles during PSIO therapy. Conversely, the small segment showed no significant internal rotation, as angles C2′-T-T′ and P2-C2′-T′ remained stable. Patients treated with active plates experienced a greater reduction in the P2-C2′-T′ angle (*p* = 0.01), suggesting center-directed rotation of the small segment in this group.

Overall, these findings highlight differences in growth patterns between the large and small segments. A possible explanation can be that the large segment likely underwent internal rotation toward the center during PSIO therapy, while the width of the anterior alveolar arch remained stable. Therefore, the monthly surface increase correlated with its monthly increase of width. By contrast, the small segment showed a correlation between surface area growth and segment length increase, with no significant internal rotation observed.

Numerous studies have examined the measurement of maxillary segments in unilateral cleft lip and palate patients. While these analyses, whether two-dimensional [6,8] or three dimensional [9,10,13], primarily relied on evaluating specific landmarks and calculating distances or angles between them, they often fell short in capturing the complexity of craniofacial structures. For a more detailed and accurate assessment of intricate anatomical features, such as those found in cleft lip and palate cases, surface measurements have proven to be superior [15]. Similarly, a narrative review by Jorge et al. [23] emphasized the importance of non-invasive diagnostic methods, including surface measurements, for assessing maxillary arches in orofacial cleft patients.

Previous surface measurement studies have predominantly utilized digitized plaster models, often evaluating data at only a few specific time points [2,12,18,19]. Ambrosio et al. [2] assessed digitized dental models of patients with UCLP at three timepoints, focusing solely on the surface area from the alveolar ridge to the intertuberosity distance. Their analysis excluded the vestibular tooth germ-bearing alveolar regions beyond the palatal gingival grooves and segment width was not measured. In alignment with our findings, their study demonstrated significant increases in jaw segment surface area and stable intercanine anterior arch width during the treatment period. However, they observed an increase in intertuberosity distances, differing from our findings. The comparison is limited because their observation period included measurements before and after cheiloplasty and one year after palatoplasty, thus incorporating surgical effects alongside growth. Bruggink et al. [19] also studied surface measurements of digitized plaster casts, focusing on the palatal area between defined anatomical landmarks while excluding vestibular alveolar regions. Their findings revealed an increase in palatal surface growth during treatment, and anterior arch width between cuspid points increased between the third and sixth months. According to our study, the distance between the tuberosities remained steady. Hoffmannova et al. [18] analyzed digitized casts from 56 UCLP patients at two time points: shortly after birth (pre-cheiloplasty) and at 10 months of age (post-cheiloplasty). They defined transverse boundaries for jaw segment surface measurements using the mesial and distal margins of the canine and molar swellings. Significant anterior segment growth reduced cleft width, while posterior segment growth was more pronounced on the non-cleft side. However, their study did not perform detailed quantitative analyses and incorporated surgical effects, making direct comparisons with our findings challenging. De Menezes and colleagues [12] assessed 96 digitized palatal casts from 32 babies with UCLP at three time points: postnatal, pre-cheiloplasty, and post-cheiloplasty. Using landmarks at the alveolar process and maxillary bone junctions, they analyzed both palatal and vestibular regions. Their postnatal surface measurements of minor (32.7 mm^2^) and major (53.2 mm^2^) segments aligned with our findings, but their study lacked detailed quantitative analyses. A multicenter study by Berkowitz et al. [14] employed a three-dimensional electromechanical palatal cast-measuring instrument to evaluate dental casts from 242 children. They observed significant total surface area growth over time, with large and small segments growing comparably. Similarly, Baek et al. [24] analyzed digitized models of 16 infants, noting significant growth in both segments, reduced cleft width, and stable posterior segment ends before surgical intervention. As our study shows, the large segment showed a different pattern of the rotational change than the small segment. Unlike our findings, they reported decreased sagittal lengths and increased widths between both segments during the PSIO period. The segmental arch width was not assessed. Detailed length measurements have so far only been carried out in studies without surface measurements. A study from Braumann et al. [25] provided insights into maxillary changes during the PSIO period. Their study on complete UCLP cases found significant cleft width reduction, constant anterior alveolar arch width, and increased posterior arch length, consistent with our results. However, their findings of increased posterior arch width and stable anterior arch length contrast with our observations.

Advances in digital scanning technology have enabled computer-aided planning and fabrication of maxillary feeding plates [26,27]. These methods enhance patient comfort and eliminate risks associated with traditional impressions, such as aspiration and tissue irritation. Additionally, intraoral scanning provides highly accurate digital impressions, free from material-related errors like distortion or tearing, improving measurement precision [28]. Surface growth evaluations offer detailed insights into jaw segment reshaping, especially in UCLP-affected regions. These measurements can detect asymmetries, monitor healing, and assess PSIO efficacy, facilitating treatment adjustments to promote symmetrical jaw development. Digital planning of feeding plates benefits significantly from monthly insights into maxillary surface changes, ensuring optimal fit and therapeutic outcomes. Precise knowledge of surface change locations and magnitudes is crucial, and advancements in digital methods could allow sectional surface change measurements, enabling detailed anterior-posterior comparisons. Since alveolar ridges house both deciduous and permanent teeth, varying dental development stages may influence area growth. Future digital anthropometric studies, particularly focusing on postoperative periods and bilateral cleft cases, are essential to deepen understanding. The measurement of individual sub-segment widths, as outlined in our study, has not yet been reported in the literature. While reproducible reference points for these measurements are still lacking, the available measurement sections are highly relevant for determining which growth direction—length or width—contributes more significantly to surface enlargement.

Differential growth patterns between maxillary segments are crucial for guiding forward planning of individualized appliances. The clinical relevance lies in determining whether adjustments should prioritize sagittal (length) or transverse (width) modifications over the course of monthly therapy. Our findings demonstrated a positive correlation between surface area and length growth in the small segment, indicating that this segment primarily expands in a sagittal direction toward the cleft. By contrast, the large segment showed greater growth in the transverse dimension, which was supported by the observed correlation between angular changes and width expansion. While the large segment also grew sagittally, it exhibited a rotational movement toward the cleft, contributing more to cleft narrowing than length extension. Consequently, the small segment facilitated length increase, while the large segment contributed to segment rotation and transverse alignment. Notably, the small segment exhibited a higher percentage of surface growth compared to the large segment. These differences in growth behavior are highly relevant for forward digital planning and timely adaptation of presurgical orthopedic appliances. In this way, maxillary plates can be designed more accurately, which help the clinician to avoid sore spots or risk misfitting the plates.

This study revealed distinct growth patterns of the large and small segments in amount and direction, underscoring the relevance of incorporating segment arch width in surface evaluations. These findings are highly relevant for clinical and scientific contexts, offering a feasible tool for digital assessments of maxillary arches in UCLP patients. Moreover, we wanted to provide a detailed month-by-month analysis of segmental surface area changes to establish a valuable basis for aiding forward planning in feeding plate production, assisting specialized centers treating patients with UCLP.

### Limitations

The limited demographic of intraoral scans and participants in this study adversely affects the generalizability of the results. Data were analyzed per protocol, which inherently carries a risk of bias. As highlighted in a study Braumann et al. [29], the success of model analyses depends not only on model quality but also on the examiner’s individual experience. To mitigate variability, intraoral scanning, digital model surface post-processing, and the setting of anatomical measurement and reference points were consistently performed by the same individual for all models.

A further limitation is the absence of standardized protocols for surface measurements in cleft lip and palate patients. While some studies have investigated surface area changes, there is a pressing need for consistent reference points, measurement techniques, and analysis criteria to ensure comparability across clinical settings. Variations in scanner types, patient cooperation, and software capabilities can significantly influence the accuracy and reliability of surface measurements.

Future innovations, such as leveraging artificial intelligence and machine learning, could address these challenges by enhancing measurement accuracy, identifying growth patterns, and predicting outcomes based on longitudinal data. These advancements hold promise for standardizing and improving the precision of surface growth analyses in clinical and research settings.

## 5. Conclusions

During PSIO therapy, the total surface area as well as the surface areas of the small and large segments of patients with UCLP increased significantly. Not only did PSIO therapy not lead to compression of the maxilla, but it also supported and guided the maxillary growth. Therefore, PSIO therapy can be recommended prior to surgical closure of cleft lip and palate.

## Figures and Tables

**Table 1:** Anatomical, constructed, and width measuring points, measuring distances, and descriptions.

	Measuring Point	Description of the Anatomical Position
**Anatomical points**	Inz	Intersection of the crest of the alveolar ridge and the line drawn from the labial frenulum to the incisive papilla
	P1/P2	Cleft edge of the crest of the alveolar ridge, large/small segment
	C2/C2′	Intersection of the gingival groove and the lateral sulcus (large/small segment)
	T/T’	The most distal point of the gingival groove (Tuberosity points)
	T-C2 vest	The most vestibular point perpendicular to the line T-C2 (large segment)
	T-C2 pal	The most palatal point perpendicular to the line T-C2 (large segment)
	C2-Inz vest	The most vestibular point perpendicular to the line C2-Inz (large segment)
	C2-Inz pal	The most palatal point perpendicular to the line C2-Inz (large segment)
	Inz-P1 vest	The most vestibular point perpendicular to the line Inz-P1 (large segment)
	Inz-P1 pal	The most palatal point perpendicular to the line Inz-P1 (large segment)
	P2-C2′ vest	The most vestibular point perpendicular to the line P2-C2′ (small segment)
	P2-C2′ pal	The most palatal point perpendicular to the line P2-C2′ (small segment)
	C2′-T’ vest	The most vestibular point perpendicular to the line C2′-T’ (small segment)
	C2′-T’ pal	The most palatal point perpendicular to the line C2′-T’ (small segment)
**Constructed** **points**	M	Center of the line connecting T and T’
	A	Perpendicular projection of Inz to the line T-T’
	Measuring distance	Description
**Alveolar cleft width**	P-P’	The line connecting P and P’; real cleft width
**Anterior and posterior arch width**	C2-C2′	Distance C2-C2′
	T-T’	Distance T-T′
	C2-M	Distance point C2 to the midpoint of line TT′ (large segment)
	C2′-M	Distance point C2′ to the midpoint of line TT′ (small segment)
**Segment length measurements**	T-C2	Distal alveolar ridge length of the large segment
	C2-Inz	Medial alveolar ridge length of the large segment
	Inz-P1	Mesial alveolar ridge length of the large segment
	P2-C2′	Mesial alveolar ridge length of the small segment
	C2′-T’	Distal alveolar ridge length of the small segment
	AKL (T-C2-Inz-P1)	Total alveolar ridge length large segment
	AKL’ (P2-C2′-T’)	Total alveolar ridge length small segment
**Segment width measurements**	T-C2 vest—T-C2 pal	Distance from the most vestibular and the most palatal point perpendicular to the distance T-C2 (large segment)/distal alveolar ridge width of the large segment
	C2-Inz vest—Inz pal	Distance from the most vestibular and the most palatal point to the distance C2-Inz (large segment)/medial alveolar ridge width of the large segment
	Inz-P1 vest—Inz-P1 pal	Distance from the most vestibular and the most palatal point to the distance Inz-P1 (large segment)/mesial alveolar ridge width of the large segment
	C2′-P2 vest—C2′-P2 pal	Distance from the most vestibular and the most palatal point to the distance C2′-P2 (small segment)/mesial alveolar ridge width of the small segment
	T’-C2′ vest—T’-C2′ pal	Distance from the most vestibular and the most palatal point to the distance T′-C2′ (small segment)/distal alveolar ridge width of the small segment
**Angle measurements**	MIA	Angle between Inz-A and Inz-M
	C2-T-T’	Curvature of the distal alveolar ridge area (large segment)
	T-C2-Inz	Curvature of the medial alveolar ridge area (large segment)
	C2-Inz-P1	Curvature of the mesial alveolar ridge area (large segment)
	P2-C2′-T’	Curvature of the mesial alveolar ridge area (small segment)
	C2′-T-T’	Curvature of the distal alveolar ridge area (small segment)

**Table 2:** Descriptive analysis of the increases in the surface areas of the jaw segments (total, small, large segment), shown as total and percentage surface increases over the whole observation period and monthly intervals. Results are stratified according to segment type (small vs. large), gender (male vs. female), and cleft side (right vs. left).

Type of Stratification			Measurement Time	N	Mean/Median	SD/IQR	*p*-Value
Total surface area			monthly (mm^2^)	49	89.01	27.20	<0.001 *
			monthly (%)	49	8.56	0.0276	<0.001 *
			overall (mm^2^)	49	363.23	157.15	<0.001 *
			overall increase (%)	49	34.89	0.1549	<0.001 *
Small segment			monthly (mm^2^)	49	38.69	13.91	<0.001 *
			monthly (%)	49	9.50	0.0357	<0.001 *
			overall (mm^2^)	49	158.33	78.48	<0.001 *
			overall increase (%)	49	39.00	0.2025	<0.001 *
Large segment			monthly (mm^2^)	49	50.32	16.85	<0.001 *
			monthly (%)	49	8.01	0.0290	<0.001 *
			overall (mm^2^)	49	204.90	87.50	<0.001 *
			overall increase (%)	49	32.43	0.1404	<0.001 *
Segment type		LS—SS	monthly (mm^2^)	49	11.62	14.66	<0.001 *^,A^
			monthly (%)	49	−1.49	0.0322	<0.001 *^,A^
			overall (mm^2^)	49	46.57	54.16	<0.001 *^,A^
			overall increase (%)	49	−6.57	0.1300	<0.001 *^,A^
Cleft side	TS	L	monthly (mm^2^)	32	89.93	24.47	0.77 **^,B^
		R	monthly (mm^2^)	17	87.29	32.46	
		L	overall (mm^2^)	32	389.10	156.62	0.11 **^,B^
		R	overall (mm^2^)	17	314.53	150.66	
	SS	L	monthly (mm^2^)	32	39.47	11.85	0.64 **^,B^
		R	monthly (mm^2^)	17	37.24	17.46	
		L	overall (mm^2^)	32	171.10	75.02	0.13 **^,B^
		R	overall (mm^2^)	17	134.27	81.41	
	LS	L	monthly (mm^2^)	32	50.46	15.28	0.94 **^,B^
		R	monthly (mm^2^)	17	50.04	19.97	
		L	overall (mm^2^)	32	217.99	89.56	0.14 **^,B^
		R	overall (mm^2^)	17	180.25	80.25	
Gender	TS	M	monthly (mm^2^)	39	91.60	27.42	0.18 **^,C^
		F	monthly (mm^2^)	10	78.93	25.06	
		M	overall (mm^2^)	39	368.11	155.46	0.69 **^,C^
		F	overall (mm^2^)	10	344.18	170.78	
	SS	M	monthly (mm^2^)	39	40.49	14.25	0.04 **^,C^
		F	monthly (mm^2^)	10	31.68	10.31	
		M	overall (mm^2^)	39	164.25	81.16	0.25 **^,C^
		F	overall (mm^2^)	10	135.24	65.49	
	LS	M	monthly (mm^2^)	39	51.10	16.93	0.53 **^,C^
		F	monthly (mm^2^)	10	47.25	17.01	
		M	overall (mm^2^)	39	203.86	82.61	0.89 **^,C^
		F	overall (mm^2^)	10	208.93	109.53	

The overall increase denotes the difference between the last and the first area measurements, whereas the monthly increase refers to the difference between the last and the first measurement divided by the number of months between the first and last measurements. The percentual increase is reported with respect to the area at the first measurement. N = sample size, SD = standard deviation, IQR = interquartile range, TS = total surface area, SS = small segment, LS = large segment, L = left side, R = right, side M = male, F = female. * Statistical significance of paired *t*-test (*p* < 0.05) ** statistical significance of two-sample *t*-test (*p* < 0.05), ^A^ LS–SS intragroup comparisons within each subcategory, ^B^ L vs. R intergroup comparisons, ^C^ M vs. F intergroup comparisons.

**Table 3:** Descriptive analysis of the increases in the surface areas of the jaw segments (total, small, large segment) compared to the first measurement and compared to the measurement of the previous month, shown as total (mm^2^) and percentage monthly surface increase.

Type of Stratification		N	Mean	SD	*p*-Value
Total surface increase compared to the first measurement	month 1 (mm^2^)	29	124.75	50.83	<0.001 *
	month 1 (%)	29	0.1218	0.0511	<0.001 *
	month 2 (mm^2^)	25	225.77	67.26	<0.001 *
	month 2 (%) n	25	0.2190	0.0685	<0.001 *
	month 3 (mm^2^)	31	293.68	102.99	<0.001 *
	month 3 (%)	31	0.2871	0.1066	<0.001 *
	month 4 (mm^2^)	28	359.49	102.59	<0.001 *
	month 4 (%)	28	0.3371	0.0994	<0.001 *
	month 5 (mm^2^)	15	488.67	99.44	<0.001 *
	month 5 (%)	15	0.4556	0.1022	<0.001 *
	month 6 (mm^2^)	9	437.29	140.49	<0.001 *
	month 6 (%)	9	0.4354	0.1407	<0.001 *
Total surface increase compared to the previous month	month 1 (mm^2^)	27	107.09	40.26	<0.001 *
	month 1 (%)	27	0.1034	0.0389	<0.001 *
	month 2 (mm^2^)	19	105.33	34.69	<0.001 *
	month 2 (%) n	19	0.0913	0.0286	<0.001 *
	month 3 (mm^2^)	17	135.39	35.64	<0.001 *
	month 3 (%)	17	0.1111	0.0326	<0.001 *
	month 4 (mm^2^)	15	104.79	0.0773	<0.001 *
	month 4 (%)	15	0.0729	0.0416	<0.001 *
	month 5 (mm^2^)	9	109.05	58.31	<0.001 *
	month 5 (%)	9	0.0766	0.0472	<0.01 *
	month 6 (mm^2^)	3	87.62	0.0227	<0.03 *
	month 6 (%)	3	0.0581	0.0129	<0.02 *
Surface increase small segment compared to the first measurement	month 1 (mm^2^)	29	58.26	27.69	<0.001 *
	month 1 (%)	29	0.1453	0.0708	<0.001 *
	month 2 (mm^2^)	25	104.30	35.32	<0.001 *
	month 2 (%) n	25	0.2562	0.0883	<0.001 *
	month 3 (mm^2^)	31	133.38	57.09	<0.001 *
	month 3 (%)	31	0.3308	0.1472	<0.001 *
	month 4 (mm^2^)	29	155.00	59.38	<0.001 *
	month 4 (%)	29	0.3761	0.1478	<0.001 *
	month 5 (mm^2^)	15	227.94	50.54	<0.001 *
	month 5 (%)	15	0.5513	0.1453	<0.001 *
	month 6 (mm^2^)	9	182.32	75.51	<0.001 *
	month 6 (%)	9	0.4604	0.1978	<0.001 *
Surface increase small segment compared to the previous month	month 1 (mm^2^)	27	49.17	23.99	<0.001 *
	month 1 (%)	27	0.1212	0.0607	<0.001 *
	month 2 (mm^2^)	19	51.14	18.34	<0.001 *
	month 2 (%) n	19	0.1090	0.0359	<0.001 *
	month 3 (mm^2^)	17	53.84	24.11	<0.001 *
	month 3 (%)	17	0.1078	0.0485	<0.001 *
	month 4 (mm^2^)	15	43.38	25.89	<0.001 *
	month 4 (%)	15	0.0821	0.0505	<0.001 *
	month 5 (mm^2^)	10	53.48	32.58	<0.001 *
	month 5 (%)	10	0.0979	0.0756	<0.01 *
	month 6 (mm^2^)	3	23.76	9.94	0.05
	month 6 (%)	3	0.0392	0.0204	0.07
Surface increase large segment compared to the first measurement	month 1 (mm^2^)	29	66.48	34.57	<0.001 *
	month 1 (%)	29	0.1073	0.0574	<0.001 *
	month 2 (mm^2^)	25	121.47	38.47	<0.001 *
	month 2 (%) n	25	0.1946	0.0639	<0.001 *
	month 3 (mm^2^)	31	160.29	59.58	<0.001 *
	month 3 (%)	31	0.2593	0.0982	<0.001 *
	month 4 (mm^2^)	28	204.79	54.07	<0.001 *
	month 4 (%)	28	0.3148	0.0873	<0.001 *
	month 5 (mm^2^)	16	256.36	62.01	<0.001 *
	month 5 (%)	16	0.3939	0.1007	<0.001 *
	month 6 (mm^2^)	9	254.97	84.78	<0.001 *
	month 6 (%)	9	0.4219	0.1371	<0.001 *
Surface increase large segment compared to the previous month	month 1 (mm^2^)	27	57.92	30.74	<0.001 *
	month 1 (%)	27	0.0924	0.0485	<0.001 *
	month 2 (mm^2^)	19	54.19	24.46	<0.001 *
	month 2 (%) n	19	0.0794	0.0355	<0.001 *
	month 3 (mm^2^)	17	81.55	27.54	<0.001 *
	month 3 (%)	17	0.1144	0.0461	<0.001 *
	month 4 (mm^2^)	15	61.39	42.90	<0.001 *
	month 4 (%)	15	0.0742	0.0472	<0.001 *
	month 5 (mm^2^)	10	57.99	35.77	<0.001 *
	month 5 (%)	10	0.0678	0–0418	<0.001 *
	month 6 (mm^2^)	4	58.06	29.49	<0.03 *
	month 6 (%)	4	0.0684	0.0312	<0.03 *

N = sample size, SD = standard deviation. * Statistical significance of paired *t*-test (*p* < 0.05).

**Table 4:** Descriptive analysis as well as comparisons of cleft reduction monthly and over the whole observation period. Results are stratified according to cleft side (right vs. left), gender (male vs. female), and type of appliance (active vs. passive).

Type of Stratification		Measurement Time	N	Mean/Median	SD/IQR	*p*-Value
Cleft width reduction		monthly (mm)	50	0.97	0.65	<0.001 ***
		monthly (%)	50	12.10	0.0823	<0.001 *
		overall (mm)	50	3.81	2.36	<0.001 *
		overall (%)	50	45.64	0.2883	<0.001 *
Cleft side	L	monthly (mm)	33	−0.91	−0.78	0.44 ****^,A^
	R	monthly (mm)	17	−1.03	0.47	
Gender	M	monthly (mm)	40	−1.00	−0.69	0.57 ****^,B^
	F	monthly (mm)	10	−0.78	−0.45	
Type of appliance	AP	monthly (mm)	30	−1.10	−0.71	0.04 ****^,C^
	PP	monthly (mm)	20	−0.75	−0.53	

N = sample size, SD = standard deviation, IQR = interquartile range, L = left side, R = right side, M = male, F = female, AP = active plate, PP = passive plate. * Statistical significance of paired *t*-test (*p* < 0.05), *** Statistical significance of Wilcoxon matched pairs test, **** statistical significance of Wilcoxon–Mann–Whitney U-test, ^A^ L vs. R intergroup comparisons, ^B^ M vs. F intergroup comparisons, ^C^ AP vs. PP intergroup comparisons.

**Table 5:** Descriptive analysis as well as comparisons of the monthly transversal measurements in UCLP patients during PSIO. Results are stratified according to segment type (small vs. large), gender (male vs. female), and cleft side (right vs. left).

Type of Stratification				Measurement Time	N	Mean/Median	SD/IQR	*p*-Value
C2-C2′	descriptive			monthly (mm)	50	−0.01	0.61	0.50 *
	Cleft side	L		monthly (mm)	33	−0.0023	0.63	0.86 **^,A^
		R		monthly (mm)	17	−0.04	0.60	
	Gender	M		monthly (mm)	40	−0.06	0.65	0.15 **^,B^
		F		monthly (mm)	10	0.18	0.39	
	Type of appliance	AP		monthly (mm)	30	−0.04	0.59	0.69 **^,C^
		PP		monthly (mm)	20	0.02	0.66	
T-T’	descriptive			monthly (mm)	50	0.14	0.59	0.08 *
	Cleft side	L		monthly (mm)	33	0.12	0.50	0.63 **^,A^
		R		monthly (mm)	17	0.21	0.73	
	Gender	M		monthly (mm)	40	0.12	0.62	0.43 **^,B^
		F		monthly (mm)	10	0.26	0.45	
	Type of appliance	AP		monthly (mm)	30	0.08	0.59	0.38 ****^,C^
		PP		monthly (mm)	20	0.16	2.22	
C2-M/ C2′-M	descriptive	LS		monthly (mm)	50	−0.09	0.44	0.17 *
		SS		monthly (mm)	50	0.07	0.35	0.18 *
	Cleft side	LS	L	monthly (mm)	33	−0.11	0.46	0.52 **^,A^
			R	monthly (mm)	17	−0.03	0.40	
		SS	L	monthly (mm)	33	0.11	0.33	0.29 **^,A^
			R	monthly (mm)	17	−0.01	0.38	
	Gender	LS	M	monthly (mm)	40	−0.11	-0.45	0.42 **^,B^
			F	monthly (mm)	10	0.01	0.39	
		SS	M	monthly (mm)	40	0.04	0.37	0.21 **^,B^
			F	monthly (mm)	10	0.17	0.25	
	Type of appliance	LS	AP	monthly (mm)	30	−0.09	0.39	1.0 **^,C^
			PP	monthly (mm)	20	−0.09	0.51	
		SS	AP	monthly (mm)	30	0.04	0.36	0.51 **^,C^
			PP	monthly (mm)	20	0.11	0.35	

N = sample size, SD = standard deviation, IQR = interquartile range, L = left side, R = right side, M = male, F = female, AP = active plate, PP = passive plate, C2-C2′ = anterior alveolar arch width, T-T’= posterior alveolar arch width, C2-M = distance point C2 to the midpoint of line TT′ (large segment), C2′-M = distance point C2′ to the midpoint of line TT′ (small segment). * statistical significance of paired *t*-test (*p* < 0.05), ** statistical significance of two-sample *t*-test (*p* < 0.05), **** statistical significance of Wilcoxon–Mann–Whitney U-test, ^A^ L vs. R intergroup comparisons, ^B^ M vs. F intergroup comparisons, ^C^ AP vs. PP intergroup comparisons.

**Table 6:** Descriptive analysis as well as comparisons of the monthly segment length and width measurements in UCLP patients during PSIO. Results are stratified according to segment type (small vs. large), gender (male vs. female), and cleft side (right vs. left).

Type of Stratification				Measurement Time	N	Mean/ Median	SD/IQR	*p*-Value
Segment length measurements								
LS	AKL	descriptive		monthly (mm)	49	1.55	0.82	<0.001 ***
				monthly (%)	49	0.0480	0.0370	<0.001 ***
		Cleft side	L	monthly (mm)	32	1.52	0.68	0.16 ****^,A^
			R	monthly (mm)	17	1.17	0.74	
		Gender	M	monthly (mm)	39	1.48	0.82	0.93 ****^,B^
			F	monthly (mm)	10	1.42	0.78	
		Type	AP	monthly (mm)	29	1.67	1.23	0.52 ****^,C^
			PP	monthly (mm)	20	1.38	0.53	
	Inz-P1	descriptive		monthly (mm)	49	0.31	0.62	<0.001 ***
				monthly (%)	49	0.0466	0.1292	<0.001 ***
	C2-Inz	descriptive		monthly (mm)	49	0.44	0.46	<0.001 *
				monthly (%)	49	0.0405	0.0424	<0.001 *
	T-C2	descriptive		monthly (mm)	49	0.79	0.66	<0.001 ***
				monthly (%)	49	0.0662	0.0630	<0.001 ***
SS	AKL’	descriptive		monthly (mm)	49	1.02	0.74	<0.001 ***
				monthly (%)	49	0.0521	0.0393	<0.001 ***
		Cleft side	L	monthly (mm)	32	0.98	0.65	0.78 ****^,A^
			R	monthly (mm)	17	1.10	0.90	
		Gender	M	monthly (mm)	39	1.07	0.81	0.57 ****^,B^
			F	monthly (mm)	10	0.83	0.31	
		Type	AP	monthly (mm)	29	1.24	0.77	0.006 ****^,C^
			PP	monthly (mm)	20	0.70	0.56	
	P2-C2′	descriptive		monthly (mm)	49	0.46	0.51	<0.001 ***
				monthly (%)	49	0.0607	0.0667	<0.001 ***
	C2′-T′	descriptive		monthly (mm)	49	0.56	0.57	<0.001 ***
				monthly (%)	49	0.0500	0.0532	<0.001 ***
Segment width measurements								
LS	B.AKL	descriptive		monthly (mm)	49	1.41	0.82	<0.001 ***
				monthly (%)	49	0.0463	0.0277	<0.001 *
		Cleft side	L	monthly (mm)	32	1.41	0.62	0.99 **^,A^
			R	monthly (mm)	17	1.41	1.13	
		Gender	M	monthly (mm)	39	1.47	0.88	0.12 ****^,B^
			F	monthly (mm)	10	1.15	0.49	
		Type	AP	monthly (mm)	29	1.41	0.91	0.57 ****^,C^
			PP	monthly (mm)	20	1.42	0.71	
	B.Inz-P1	descriptive		monthly (mm)	49	0.23	0.42	<0.001 ***
				monthly (%)	49	0.0346	0.0670	<0.001 ***
	B.C2-Inz	descriptive		monthly (mm)	49	0.46	0.40	<0.001 *
				monthly (%)	49	0.0483	0.0427	<0.001 *
	B.T-C2	descriptive		monthly (mm)	49	0.72	0.32	<0.001 *
				monthly (%)	49	0.0556	0.0289	<0.001 ***
SS	B.AKL’	descriptive		monthly (mm)	49	1.05	0.80	<0.001 ***
				monthly (%)	49	0.0468	0.0368	<0.001 ***
		Cleft side	L	monthly (mm)	32	0.97	0.57	0.40 ****^,A^
			R	monthly (mm)	17	1.21	1.11	
		Gender	M	monthly (mm)	39	1.06	0.83	0.71 ****^,B^
			F	monthly (mm)	10	1.00	0.68	
		Type	AP	monthly (mm)	29	1.14	0.70	0.58 ****^,C^
			PP	monthly (mm)	20	0.93	0.93	
	B.P2-C2‘	descriptive		monthly (mm)	49	0.50	0.36	0.001 ***
				monthly (%)	49	0.0551	0.0377	0.001 *
	B.C2′-T’	descriptive		monthly (mm)	49	0.55	0.59	0.001 ***
				monthly (%)	49	0.0421	0.0470	0.001 ***

N = sample size, SD = standard deviation, IQR = interquartile range, LS = large segment, SS = small segment, L = left side, R = right side, M = male, F = female, PP = passive plate, AP = active plate. Segment length measurements: AKL (T-C2-Inz-P1) = total alveolar ridge length large segment, Inz-P1 = mesial alveolar ridge length of the large segment, C2-Inz = medial alveolar ridge length of the large segment, T-C2 = distal alveolar ridge length of the large segment; AKL’ (P2-C2′-T’) = total alveolar ridge length small segment, P2-C2′ = mesial alveolar ridge length of the small segment, C2′-T’ = distal alveolar ridge length of the small segment. Segment width measurements: B.AKL (T-C2-Inz-P1) = total alveolar ridge width large segment, B.Inz-P1 (Inz-P1 vest—Inz-P1 pal) = mesial alveolar ridge width of the large segment, B.C2-Inz (C2-Inz vest—Inz pal ) = medial alveolar ridge width of the large segment, B.T-C2 (T-C2 vest—T-C2 pal ) = distal alveolar ridge width of the large segment; B.AKL’ (P2-C2′-T’) = total alveolar ridge width small segment, B.P2-C2′ (C2′-P2 vest—C2′-P2 pal) = mesial alveolar ridge width of the small segment, B.C2′-T’ (T′-C2′ vest—T′-C2′ pal) = distal alveolar ridge width of the small segment. * statistical significance of paired *t*-test (*p* < 0.05), ** statistical significance of two-sample *t*-test (*p* < 0.05), *** statistical significance of Wilcoxon matched pairs test, **** statistical significance of Wilcoxon–Mann–Whitney U-test, ^A^ L vs. R intergroup comparisons, ^B^ M vs. F intergroup comparisons, ^C^ AP vs. PP intergroup comparisons.

**Table 7:** Correlations between monthly surface change with length and width change of the small and large segment. Results are stratified according to cleft side (right vs. left) and gender (male vs. female).

Surface Change				Length Change Small Segment			Width Change Small Segment		
		Observation Period	N	r	95% CI	*p*-Value	r	95% CI	*p*-Value
Small segment		monthly (mm^2^)	49	0.32	0.05–0.55	0.02 *	0.21	−0.06–0.46	0.13
		monthly (%)	49	0.32	0.05–0.55	0.02 *	0.21	−0.07–0.46	0.14
Cleft side	L	monthly (mm^2^)	32	0.004	−0.34–0.35	0.98	0.39	0.05–0.65	0.02 *
	R	monthly (mm^2^)	17	0.65	0.25–0.86	0.004 *	0.12	−0.38–0.56	0.64
Gender	M	monthly (mm^2^)	39	0.30	−0.006–0.569	0.05	0.20	−0.11–0.48	0.20
	F	monthly (mm^2^)	10	0.34	−0.36–0.79	0.33	0.26	−0.43–0.76	0.46
Surface change				Length change large segment			Width change large segment		
		observation period	N	r	95% CI	*p* value	r	95% CI	*p* value
Large segment		monthly (mm^2^)	49	0.24	−0.03–0.49	0.08	0.36	0.09–0.58	0.009 *
		monthly (%)	49	0.32	0.05–0.56	0.02 *	0.42	0.16–0.63	0.002 *
Cleft side	L	monthly (mm^2^)	32	−0.008	−0.35–0.34	0.96	0.07	−0.28–0.41	0.69
	R	monthly (mm^2^)	17	0.42	−0.07–0.75	0.09	0.61	0.18–0.84	0.008 *
Gender	M	monthly (mm^2^)	39	0.31	−0.003–0.57	0.05	0.44	0.15–0.66	0.004 *
	F	monthly (mm^2^)	10	−0.25	−0.76–0.45	0.48	−0.28	−0.77–0.41	0.42

N = sample size, r = Pearson’s correlation coefficient, 95% CI = 95 percentile confidence interval, L = left side, R = right side M = male, F = female. * Pearson’s correlation test.

**Table 8:** Descriptive analysis as well as comparisons of the monthly vertical changes in UCLP patients during PSIO. Results are stratified according to segment type (small vs. large), gender (male vs. female) and cleft side (right vs. left).

Type of Stratification				Measurement Time	N	Mean/ Median	SD/IQR	*p* Value
LS	P1-TT′Inz	descriptive		monthly (mm)	50	0.02	0.43	0.47 ***
				monthly (%)	50	1.14	0.0852	0.36 ***
		Cleft side	L	monthly (mm)	33	0.02	0.35	0.88 **^,A^
			R	monthly (mm)	17	0.002	0.57	
		Gender	M	monthly (mm)	40	0.03	0.44	0.72 **^,B^
			F	monthly (mm)	10	-0.02	0.39	
		Type	AP	monthly (mm)	30	0.12	0.36	0.06 **^,C^
			PP	monthly (mm)	20	-0.13	0.49	
	C2-TT′Inz	descriptive		monthly (mm)	50	0.24	0.25	<0.001 *
				monthly (%)	50	57.20	5.1824	0.05 ***
		Cleft side	L	monthly (mm)	33	0.27	0.24	0.37 **^,A^
			R	monthly (mm)	17	0.20	0.26	
		Gender	M	monthly (mm)	40	0.24	0.26	089 **^,B^
			F	monthly (mm)	10	0.25	0.18	
		Type	AP	monthly (mm)	30	0.24	0.23	085 **^,C^
			PP	monthly (mm)	20	0.25	0.27	
SS	P2-TT′Inz	descriptive		monthly (mm)	50	0.23	0.46	0.0002 ***
				monthly (%)	50	4.37	0.0980	0.0002 ***
		Cleft side	L	monthly (mm)	33	0.26	0.37	0.58 **^,A^
			R	monthly (mm)	17	0.16	0.68	
		Gender	M	monthly (mm)	40	0.22	0.54	0.65 **^,B^
			F	monthly (mm)	10	0.26	0.20	
		Type	AP	monthly (mm)	30	0.39	0.45	0.0012 ****^,C^
			PP	monthly (mm)	20	−0.01	0.46	
	C2′-TT′Inz	descriptive		monthly (mm)	50	0.23	0.25	<0.001 ***
				monthly (%)	50	14.17	0.4579	<0.001 ***
		Cleft side	L	monthly (mm)	33	0.23	0.28	0.66 ****^,A^
			R	monthly (mm)	17	0.19	0.30	
		Gender	M	monthly (mm)	40	0.20	0.30	0.12 ****^,B^
			F	monthly (mm)	10	0.29	0.21	
		Type	AP	monthly (mm)	30	0.24	0.34	0.67 ****^,C^
			PP	monthly (mm)	20	0.18	0.17	

N = sample size, SD = standard deviation, IQR = interquartile range, LS = large segment, SS = small segment, L = left side, R = right side, M = male, F = female, PP = passive plate, AP = active plate. P1-TT′Inz = vertical distance of the Point P1 to the reference plane (TT′Inz) (large segment), P2-TT′Inz = vertical distance of the Point P2 to the reference plane (TT′Inz) (small segment), C2-TT′Inz = vertical distance of the Point C2 to the reference plane (TT′Inz) (large segment), C2′-TT′Inz = vertical distance of the Point C2′ to the reference plane (TT′Inz) (small segment). * statistical significance of paired *t*-test (*p* < 0.05), ** statistical significance of two-sample *t*-test (*p* < 0.05), *** statistical significance of Wilcoxon matched pairs test, **** statistical significance of Wilcoxon–Mann–Whitney U-test, ^A^ L vs. R intergroup comparisons, ^B^ M vs. F intergroup comparisons, ^C^ AP vs. PP intergroup comparisons.

**Table 9:** Descriptive analysis as well as comparisons of the monthly angular measurements in UCLP patients during PSIO. Results are stratified according to segment type (small vs. large), gender (male vs. female), and cleft side (right vs. left).

Type of Stratification				Measurement Time	N	Mean/ Median	SD/IQR	*p*-Value
Length Measurements								
MIA		descriptive		monthly (mm)	50	−1.61	1.43	<0.001 ***
				monthly (%)	50	−0.0848	0.1152	<0.001 ***
		Cleft side	L	monthly (mm)	33	−0.09	0.13	0.26 ****^,A^
			R	monthly (mm)	17	−0.08	0.08	
		Gender	M	monthly (mm)	40	−1.51	1.32	0.97 ****^,B^
			F	monthly (mm)	10	−1.99	1.83	
		Type	AP	monthly (mm)	30	−0.09	0.05	0.55 ***
			PP	monthly (mm)	20	−0.08	0.18	
LS	C2-T-T′	descriptive		monthly (mm)	50	−0.49	1.53	0.03 *
				monthly (%)	50	−0.0051	0.0171	0.04 *
		Cleft side	L	monthly (mm)	33	−0.61	1.77	0.33 **^,A^
			R	monthly (mm)	17	−0.24	0.92	
		Gender	M	monthly (mm)	40	−0.51	1.55	0.46 ****^,B^
			F	monthly (mm)	10	−0.39	1.56	
		Type	AP	monthly (mm)	30	−0.38	1.39	0.60 **^,C^
			PP	monthly (mm)	20	−0.64	1.74	
	T-C2-Inz	descriptive		monthly (mm)	50	8.86	65.17	0.32 ***
				monthly (%)	50	0.0592	0.4333	0.36 ***
		Cleft side	L	monthly (mm)	33	13.24	80.25	0.50 ****^,A^
			R	monthly (mm)	17	0.37	3.18	
		Gender	M	monthly (mm)	40	11.44	72.81	0.27 ****^,B^
			F	monthly (mm)	10	−1.43	1.73	
		Type	AP	monthly (mm)	30	15.13	84.08	0.35 ****^,C^
			PP	monthly (mm)	20	−0.53	2.59	
	C2-Inz-P1	descriptive		monthly (mm)	50	−1.27	3.04	0.003 ***
				monthly (%)	50	−0.01	0.02	0.004 ***
		Cleft side	L	monthly (mm)	33	−0.87	2.62	0.45 ****^,A^
			R	monthly (mm)	17	−2.06	3.68	
		Gender	M	monthly (mm)	40	−1.46	3.13	0.64 ****^,B^
			F	monthly (mm)	10	−0.52	2.26	
		Type	AP	monthly (mm)	30	−1.59	1.75	0.09 ****^,C^
			PP	monthly (mm)	20	−0.80	4.32	
SS	C2′-T-T′	descriptive		monthly (mm)	50	0.36	1.65	0.06 ***
				monthly (%)	50	0.0052	0.0215	0.09 *
		Cleft side	L	monthly (mm)	33	0.53	1.53	0.34 **^,A^
			R	monthly (mm)	17	0.03	1.88	
		Gender	M	monthly (mm)	40	0.34	1.80	0.79 **^,B^
			F	monthly (mm)	10	0.45	0.93	
		Type	AP	monthly (mm)	30	0.61	1.65	0.19 ****^,C^
			PP	monthly (mm)	20	−0.01	1.63	
	P2-C2′-T′	descriptive		monthly (mm)	50	0.59	3.53	0.06 ***
				monthly (%)	50	0.0043	0.2343	0.05 ***
		Cleft side	L	monthly (mm)	33	1.34	2.88	0.07 **^,A^
			R	monthly (mm)	17	−0.87	4.26	
		Gender	M	monthly (mm)	40	0.49	3,79	0.60 ****^,B^
			F	monthly (mm)	10	0.99	2.29	
		Type	AP	monthly (mm)	30	−0.27	3.04	0.01 ****^,C^
			PP	monthly (mm)	20	1.89	3.89	

N = sample size, SD = standard deviation, IQR = interquartile range, LS = large segment, SS = small segment, L = left side, R = right side, M = male, F = female, PP = passive plate, AP = active plate. MIA = angle between Inz-A and Inz-M, C2-T-T′ = curvature of the distal alveolar ridge area (large segment), T-C2-Inz = curvature of the medial alveolar ridge area (large segment), C2-Inz-P1 = curvature of the mesial alveolar ridge area (large segment), P2-C2′-T′ = curvature of the mesial alveolar ridge area (small segment), C2′-T-T′ = curvature of the distal alveolar ridge area (small segment). * statistical significance of paired *t*-test (*p* < 0.05), ** statistical significance of two-sample *t*-test (*p* < 0.05), *** statistical significance of Wilcoxon matched pairs test, **** statistical significance of Wilcoxon–Mann–Whitney U-test, ^A^ L vs. R intergroup comparisons, ^B^ M vs. F intergroup comparisons, ^C^ AP vs. PP intergroup comparisons.

## Data Availability

The data are available upon request from the corresponding author.

## Abbreviations

The following abbreviations are used in this manuscript:

UCLP	Unilateral Cleft Lip and Palate
PSIO	Pre-Surgical Infant Orthopedics
